# Altitude and metabolic syndrome in China: Beneficial effects of healthy diet and physical activity

**DOI:** 10.7189/jogh.13.04061

**Published:** 2023-06-30

**Authors:** Junmin Zhou, Ruifeng He, Zhuozhi Shen, Yan Zhang, Xufang Gao, Dejiquzong, Xiong Xiao, Tao Zhang, Dan Yang, Yufei Wang, Huan Song, Yuming Guo, Shanshan Li, Gongbo Chen, Jianzhong Yin, Xing Zhao, China Multi-Ethnic Cohort (CMEC) collaborative group

**Affiliations:** 1 West China School of Public Health and West China Fourth Hospital, Sichuan University, Chengdu, China; 2 Tibet Center for Disease Control and Prevention, Lhasa, China; 3 Chongqing Municipal Center for Disease Control and Prevention, Chongqing, China; 4 School of Public Health, Guizhou Medical University, Guiyang, China; 5 Chengdu Center for Disease Control & Prevention, Chengdu, China; 6 Tibet University, Lhasa, China; 7 West China Biomedical Big Data Center, West China Hospital, Sichuan University, Chengdu, China; 8 Med-X Center for Informatics, Sichuan University, Chengdu, China; 9 Climate, Air Quality Research Unit, School of Public Health and Preventive Medicine, Monash University, Melbourne, Australia; 10 School of Public Health, Kunming Medical University, Kunming, China; 11 Baoshan College of Traditional Chinese Medicine, Baoshan, China; * Joint first authorship.

## Abstract

**Background:**

The correlation between altitude and metabolic syndrome has not been extensively studied, and the mediation effects of diet and physical activity remain unclear. We evaluated the cross-sectional correlations between altitude and metabolic syndrome and the possible mediation effects of diet and physical activity in China.

**Methods:**

We included 89 485 participants from the China Multi-Ethnic Cohort. We extracted their altitude information from their residential addresses and determined if they had metabolic syndrome by the presence of three or more of the following components: abdominal obesity, reduced high-density lipoprotein cholesterol (HDL-C), elevated triglycerides, elevated glucose, and high blood pressure at recruitment. We conducted multivariable logistic regression and mediation analyses for all and separately for Han ethnic participants.

**Results:**

The participants had a mean age of 51.67 years and 60.56% were female. The risk difference of metabolic syndrome was -3.54% (95% confidence interval (CI) = -4.24, -2.86) between middle and low altitudes, -1.53% (95%CI = -2.53, -0.46) between high and low altitudes, and 2.01% (95% CI = 0.92, 3.09) between high and middle altitudes. Of the total estimated effect between middle and low altitude, the effect mediated by increased physical activity was -0.94% (95% CI = -1.04, -0.86). Compared to low altitude, the effects mediated by a healthier diet were -0.40% (95% CI = -0.47, -0.32) for middle altitude and -0.72% (95% CI = -0.87, -0.58) for high altitude. Estimates were similar in the Han ethnic group.

**Conclusions:**

Living at middle and high altitudes was significantly associated with lower risk of metabolic syndrome compared to low altitude, with middle altitude having the lowest risk. We found mediation effects of diet and physical activity.

Metabolic syndrome is a cluster of risk factors for cardiovascular diseases, metabolic diseases, and overall mortality. It affects 20% to 30% of adults in most countries and poses a high disease burden globally [1].

In 2010, 842 million people (12% of the total global population) resided at 1500 m or more above sea level, with 36% of them concentrated in East and South Asia [2]. However, due to the rugged geographical environment and historical isolation, health issues in this population have been understudied [3,4]. Furthermore, most of current studies focusing on altitude and health are limited by their ecological design [5]. The few studies that used individual data were constricted by either small samples or relatively low variability of altitude, potentially impacting the robustness of their results. Several studies examining the effects of living at high altitudes on individual components of metabolic syndrome found that living at high altitude is inversely associated with obesity [6-8] and diabetes or glucose [9,10], but positively related to blood pressure [11] and dyslipidaemia [12]. However, these studies focused on one specific metabolic outcome as opposed to comprehensive indicators which may better reflect people’s cardiometabolic health profiles.

Using data from a cross-sectional survey, we aimed to examine the correlation between altitude and metabolic syndrome in Southwest China, an area which covers a wide range of altitudes and peoples including Tibetans, one of the three major high-altitude communities in the world [13]. We hypothesised that altitude would have an inverse correlation with metabolic syndrome.

The mechanisms underlying this correlation, if present, remain unclear, and might be affected by diet and physical activity (PA), which are key modifiable lifestyles that vary widely at different altitude levels [14]. Therefore, our secondary aim was to investigate the mediating effects of diet and PA on the correlation between altitude and metabolic syndrome. We hypothesised that both diet and PA would mediate the correlation between altitude and metabolic syndrome.

## METHODS

### Population

The source of data was a baseline survey of the China Multi-Ethnic Cohort (CMEC) study [15]. The CMEC is a community-based cohort study which included 99 556 permanent residents from the Tibetan, Yi, Miao, Bai, Dong, Bouyei, and Han ethnicities in Southwest China. The study complied with the Declaration of Helsinki and received ethical approval from the Sichuan University Medical Ethical Review Board (K2016038, K2020022). All participants provided written informed consent prior to data collection.

We used data from 89 485 participants aged 30-79 years (n = 98 513), with a valid home address (n = 97 728), and with no missing values for all included variables (n = 89 485). The participants were not involved in the design or conduct of research.

### Measurements

#### Assessment of exposure

**Figure 1:**
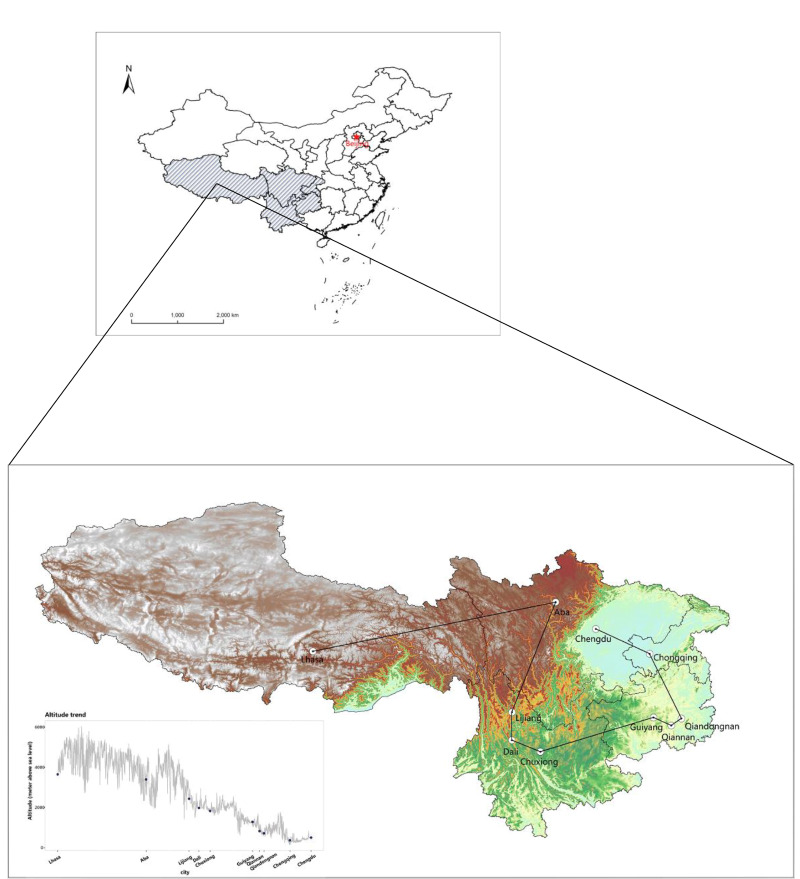
Spatial distribution of study sites.

We downloaded global Shuttle Radar Topography Mission (SRTM) 4 elevation data for China at a spatial resolution of three arc-seconds (approximately 90 m) from the Consultative Group on International Agricultural Research – Consortium for Spatial Information (http://srtm.csi.cgiar.org/). We extracted the participants’ altitude information from the SRTM data according to their residential addresses. They lived at altitudes ranging from 7 m to 5346 m (**Figure 1**), so we divided them into low altitude (0-1499 m), middle altitude (1500-2999 m), and high altitude (≥3000 m) groups [6,12].

#### Ascertainment of outcome

We used the National Cholesterol Education Program – Adult Treatment Panel III criteria to assess metabolic syndrome [16]. We defined abdominal obesity as a waist circumference ≥90 cm for men and ≥85 cm for women [17], reduced HDL-C as a level <1.03 mmol/L (<40 mg/dL) in men or 1.29 mmol/L (<50 mg/dL) in women, or using drug treatment for reduced HDL-C [16]. We defined elevated triglycerides as fasting triglycerides level ≥1.7 mmol/L (150 mg/dL), or being on drug treatment for elevated triglycerides [16] and elevated glucose as a fasting plasma glucose level ≥5.6 mmol/L (100 mg/dL) or using drug treatment for elevated glucose [16]. We classified high blood pressure as ≥130/85 mm Hg or using antihypertensive drug treatment in a patient with a history of hypertension [16]. We considered the presence of three or more of these components as metabolic syndrome [16].

Trained technicians obtained waist circumference measurements by placing a soft non-stretchable tape placed midway between the participants’ iliac crest and the lowest rib, to the nearest 0.1 cm. The participants wore only light clothes and fasted overnight (at least eight hours). Venous blood samples were collected on site. We measured blood lipids (including HDL-C and triglycerides) and glucose using an AU5800 Automated Chemistry Analyzer (Beckman Coulter Commercial Enterprise, Shanghai, China). Trained technicians, following the American Heart Association’s standardised protocol [18], measured participants’ blood pressure using electronic sphygmomanometers, which were calibrated before measurement. We averaged three blood pressure readings to calculate systolic and diastolic blood pressures.

#### Assessment of mediators

We assessed dietary patterns using a food frequency questionnaire regarding participants’ food consumption in the past year. We used the Dietary Approaches to Stop Hypertension (DASH) guidelines to determine adherence to a healthy diet, modifying the DASH score by replacing non-fat and low-fat dairy by full-fat dairy products [19], which are universally consumed in China. According to a randomised controlled trial [19], the modified DASH diet was more likely to reduce cardiometabolic risks. We excluded the food group component of sweetened beverages since the regular consumption of sweetened beverages was as low as 7.2% in our sample. For each of the remaining seven food group components of DASH, we categorised food group consumption into quintiles and scored all participants from 1 to 5 based on their intake ranking. Thus, the DASH score for each participant ranged between 7 and 35 (minimal to maximal adherence).

PA considered participants’ occupational, traffic, chores, and leisure time activities. We obtained both PA intensity (quantified as using the corresponding metabolic equivalent values (MET) [20,21]) and duration during the preceding year. We calculated the product of PA intensity (MET) and duration (hours) as the volume of activity (MET-h), grouping all participants into three categories based on terciles of the PA volume: low (0-14.9 MET-h/d), moderate (15.0-31.6 MET-h/d), and high (≥31.7 MET-h/d). We used both continuous PA and PA for data analysis.

#### Assessment of covariates

We collected information on covariates (i.e. age, sex, marital status, educational level, income, alcohol consumption, smoking, and passive smoking) at recruitment through a questionnaire. We did not include body mass index (BMI), since the metabolic syndrome definition included abdominal obesity.

### Statistical analysis

To simultaneously compare the prevalence of metabolic syndrome between the three altitude groups, we used the likelihood ratio test to compare the deviance between two nested models, with or without the altitude variable. A logistic regression model was used to examine the correlation between altitude and metabolic syndrome. We presented the results as risk difference (RD) using average predictive comparison with corresponding 95% confidence intervals (CIs) in altitude [22]. RD estimates the expected difference in metabolic syndrome associated with different altitude levels. In linear models with no interactions, this is identical to the coefficient estimates, while in generalised linear models, it allows interpretation of the original scale of the response variable, which, in the case of logistic regression, is the probability scale rather than the odds.

We performed stratified analysis by age (30-60 years, 60-70 years, 70-79 years), sex, marital status (did not co-habit, co-habited), educational level (illiteracy, primary school, junior high school, high school or above), income (<20 000, 20 000-59 999, ≥60 000, in RMB Yuan), smoking status (never, current, former), passive smoking (no, yes), and alcohol consumption (0 g/d, (0, 5)g/d, ≥5g/d).

We performed mediation analysis of dietary patterns and PA. First, we examined the interaction by including a product term for altitude and diet in the model and performing a test for altitude and PA. We tested the mediators one at a time, using the mediation package to estimate the direct, indirect, and total effects and the proportion mediated [23]. To examine the robustness of the estimates for ethnicity, we conducted sensitivity analyses by repeating all the above analyses for the Han ethnic group.

We adjusted all multivariable models for all covariates unless stated otherwise. We did not adjust for BMI, because it highly correlated with waist circumference in the metabolic syndrome (correlation coefficient (*r*) = 0.78; *P* < 0.0001) based on previous literature [24]. The correlation was statistically significant if the two-sided *P*-value was <0.05. We performed all statistical analyses using R (version 3.6.1, R Foundation for Statistical Computing, Vienna, Austria).

### Patient and public involvement

Patients or the public were not involved in the study.

## RESULTS

### Participants characteristics

The mean age of the 89 485 participants was 51.67 years (range = 30-79), and 60.56% were female (**Table 1**); 8701 were from high, 21 395 from middle, and 59 389 from low altitude settings.

**Table 1:** Baseline characteristics of the included participants according to different altitude groups*

Characteristic	All participants (n = 89 485)	High altitude (n = 8701)	Middle altitude (n = 21 395)	Low altitude (n = 59 389)	*P* value
**Age in years, mean (SD)**	51.67 (11.48)	48.87 (11.19)	52.90 (10.42)	51.63 (11.81)	<0.001
**Sex**					
Male	39.44	39.32	31.90	42.04	<0.001
Female	60.56	60.68	68.10	57.96	
**Marital status**					
Did not cohabit	11.22	12.44	10.30	11.35	<0.001
Cohabited	88.78	87.56	89.70	88.65	
**Educational level**					
Illiteracy	26.54	65.77	27.42	20.06	<0.001
Primary school	25.35	25.04	38.35	20.37	
Junior high school	25.59	5.51	26.32	29.09	
High school or above	22.52	3.68	7.91	30.47	
**Income in yuan†**					
<20 000	35.70	50.75	43.26	30.52	<0.001
20 000-59 999	36.12	37.11	42.05	34.11	
≥60 000	28.18	12.14	14.69	35.37	
**Smoking status**					
Never	75.13	83.71	77.21	73.32	<0.001
Current	19.84	11.39	19.82	20.90	
Former	5.04	4.90	2.96	5.79	
**Passive smoking**					
No	51.85	84.97	46.45	48.89	<0.001
Yes	48.15	15.03	53.55	51.11	
**Alcohol consumption in g/d**					
0	57.71	81.28	74.54	48.31	<0.001
(0, 5)	29.79	14.63	15.92	36.96	
≥5	12.50	4.08	9.55	14.73	
**Physical activity (MET-h/d), mean (SD)†**	26.05 (18.25)	20.62 (16.95)	33.22 (19.04)	24.29 (16.55)	<0.001
**Dietary pattern (DASH score), mean (SD)**	20.48 (4.41)	20.56 (3.55)	20.72 (3.89)	20.39 (4.65)	<0.001
**Metabolic syndrome**					
No	74.77	76.43	78.18	73.30	<0.001
Yes	25.23	23.57	21.82	26.70	

SD – standard deviation, DASH – Dietary Approaches to Stop Hypertension, MET – metabolic equivalent

*Data are percentages unless otherwise indicated. Data were age adjusted where appropriate. One-way analysis of variance and χ^2^ tests were used to produce *P*-values.

†As of January 2021, the exchange rate was approximately 6.46 Yuan per US$.

### Correlation between altitude and metabolic syndrome

The prevalence of metabolic syndrome was higher in the low altitude group (26.70%) than in the high (23.57%) and middle altitude (21.82%) groups (**Table 1**). The RD of metabolic syndrome was -3.54% (95% CI = -4.24, -2.86) between the middle and the low altitude groups, and -1.53% (95% CI = -2.53, -0.46) between the high and the low altitude groups, while the RD between the high and the middle altitude groups was 2.01% (95% CI = 0.92, 3.09) after adjusting for potential confounders (**Table 2**).

**Table 2:** Correlation between altitude and metabolic syndrome*

				Likelihood ratio test of altitude‡		
**Altitude**	**Risk difference, %**	**95% CI, %**	***P*-value**	**Deviance**	**df**	***P*-value**
Middle to low	-3.54	(-4.24, -2.86)	<0.001†	102.79	2.00	<0.001
High to low	-1.53	(-2.53, -0.46)	0.016†			
High to middle	2.01	(0.92, 3.09)	<0.001†			

CI – confidence interval, df – degrees of freedom

*Analyses were adjusted for age, sex, marital status, education, income, smoking status, passive smoking, alcohol consumption (continuous), physical activity, and dietary pattern.

**†**The difference of multiple comparison was significant, which we corrected using the Bonferroni method, α = 0.05/3.

‡To simultaneously compare the prevalence of metabolic syndrome between three altitude groups, we conducted the likelihood ratio test to compare the deviance between two nested models, which were models with or without altitude variable.

#### Mediation effects of diet and PA

**Figure 2:**
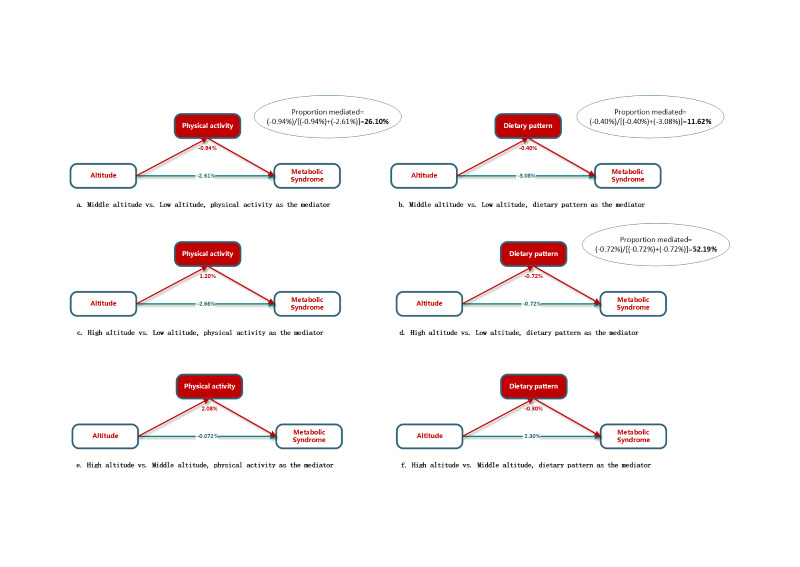
Mediation analyses of altitude (binary) and mediators of physical activity and dietary pattern (continuous) on metabolic syndrome. Analyses were adjusted for age, sex, marital status, education, income, smoking status, passive smoking, and alcohol consumption (continuous).

The interactions between altitude and diet and altitude and PA were not significant (*P* > 0.05). We detected mediation effects of diet and PA. Assuming underlying assumptions of the mediation analyses hold, the correlation between altitude and metabolic syndrome can be partly explained by increased levels of PA only when comparing middle altitude to low altitude. Of the total estimated effect, the effect and proportion mediated were -0.94% (95% CI = -1.04, -0.86) and 26.10% (95% CI = 22.05, 34.92), respectively (**Figure 2** and Table S1 in the **Online Supplementary Document**). For dietary patterns, the correlation could have been partly explained by higher DASH scores when comparing middle and high altitudes to low altitude. The effect mediated and proportion mediated were -0.40% (95% CI = -0.47, -0.32) and 11.62% (95% CI = 8.30, 14.94) for middle altitude vs low altitude, respectively, and -0.72% (95% CI = -0.87, -0.58) and 52.19% (95% CI = 26.38, 100.00) for high altitude vs low altitude, respectively (the 100.00% was truncated from 167.14% (explanations for proportion mediated in the mediation analysis in the **Online Supplementary Document**)).

Results of stratified analyses are presented in the Figures S1A-S1c in the **Online Supplementary Document**. The prevalence of the five components of metabolic syndrome and their possible combinations (three out of five components) at different altitudes are presented in the Tables S2 and S3 **Online Supplementary Document**. The sensitivity analysis including only Han people from middle and low altitudes showed that middle altitude Han people had a lower risk of metabolic syndrome than their low altitude counterparts (RD = -6.58%; 95% CI = -7.48, -5.69). The effect and proportion of the total estimated effect mediated by PA were -1.28% (95% CI = -1.51, -1.02) and 19.22% (95% CI = 14.55, 24.30), respectively, while the effect and proportion mediated by dietary pattern were -0.31% (95% CI = -0.37, -0.24) and 4.75% (95% CI = 3.40, 5.85), respectively (Table S5-S8 and Figure S2 and S3 in the **Online Supplementary Document**).

## DISCUSSION

In our study involving nearly 90 000 adults across areas with a wide range of altitudes in Southwest China, living at middle and high altitudes was significantly associated with lower risk of metabolic syndrome compared to low altitude, with middle altitude having the lowest risk. Notably, diet mediated such correlations in both middle and high altitudes when compared to low altitude, while PA mediated these correlations in middle altitude vs low altitude. If these findings are largely causal, they would not only help explain varied incidence of cardiometabolic diseases among individuals living at different altitudes, but also have public health implications for behavioural interventions at lower altitudes.

### Comparison with other studies

There is limited evidence on the correlation between altitude and metabolic syndrome, as most studies focused on metabolic profiles among people living at high altitudes [25] without comparing metabolic syndrome at different altitudes. Only one cross-sectional study involving 260 Ecuadorians has shown an inverse correlation between metabolic syndrome and altitude [26]. A few epidemiological studies examined altitude and specific metabolic outcome and found that higher altitude is protective against obesity and diabetes [6-10], but harmful to blood pressure and blood lipids [11,12]. Such findings were primarily based on altitudes around 2000 m, which is comparable to our middle altitude and in line with our findings that this group was less likely to have metabolic syndrome, large waist circumference, reduced HDL-C, and elevated fasting glucose, while being more likely to have elevated triglycerides than the low altitude group.

Although no studies have examined the correlation between high altitude and metabolic syndrome, two studies have shown that, high altitude (≥3000 m) had a similar but stronger inverse correlation with diabetes and central obesity compared to low altitude than middle altitude did [6,9]. A study on diabetes [9] was consistent with our findings of elevated fasting glucose, while a central obesity study [6] showed found the lowest prevalence of large waist circumference at high altitude, where we found it to be highest. This discrepancy could be due to the different ethnicities and different lifestyle behaviours between the two study populations (Tibetans vs Peruvians). Our findings that high altitude compared to middle altitude had similar but smaller effects on metabolic syndrome advance our current knowledge, but require further verification in future studies.

### Potential mechanism

The potential mechanism underlying these correlations remains unclear. However, studies have proposed that diverse factors, such as ethnicity, temperature, chronic hypoxia, and lifestyle behaviours might play an important role [12,27]. First, people residing at high or middle altitudes tend to be ethnically distinct from those living at low altitudes [27], which might have confounded the correlations. Second, metabolic expenditures are increased to cope with extreme temperatures caused by elevated altitude [8,28], meaning exertions at higher altitudes may represent greater exercise and in turn promote heart health [29]. Third, every contained less oxygen in higher altitudes than in lower altitude because of the decreasing barometric pressure [30]. Exposure to chronic hypoxia may modulate plasma leptin levels through hypoxia-inducible factor 1, and thus produces negative feedback on appetite to prevent obesity [31]. Fourth, lifestyle behaviours differed across altitudes, and might have moderated the correlations, which we examined and confirmed through our mediation analyses. We found that dietary pattern tended to be healthier and PA levels higher with increasing altitudes (Table S4 in the **Online Supplementary Document**). For instance, coarse grain was consumed more at high altitude, while animal oil was more prevalent at low altitude [32]. Such different dietary patterns and PA levels may account for the observed differences in metabolic syndrome at different altitudes. One exception is the lower PA at high altitude than at middle altitude. This exception was consistent with and might explain our finding that risk of metabolic syndrome was higher at high altitude than at middle altitude.

### Limitations and strengths

This study has several limitations. First, the cross-sectional design does not allow us to infer causality, so reverse causality could be problematic. For example, it could not be ruled out for diet or physical activity. However, all participants were permanent residents, so the reverse causality between altitude and metabolic syndrome would be unlikely. Second, some information was based on self-report, allowing for possible recall bias, especially for variables based on participants’ long-term memory such as history of hypertension or diabetes. Third, ethnicity might have confounded our findings, as we included participants from different ethnicities residing at different altitudes. Specifically, the high altitude group consisted of Tibetans, the middle altitude group included Han, Bai, and Yi, and the low altitude group consisted of Han, Dong, Miao, and Bouyei ethnicities. To minimise such confounding effects, we conducted a separate analysis including only Han people from middle and low altitudes (Tables S5-S8 and Figures S2 and S3 in the **Online Supplementary Document**). The findings showed that middle altitude Han people had a lower risk of metabolic syndrome than their low altitude counterparts, implying that the observed effects of altitude were unlikely to be confounded by ethnicity.

Despite these limitations, our study is unique. Our data were based on a large sample with a wide range of altitudes, which allowed us to explore the valid effects of high altitude. Moreover, as opposed to focusing on specific cardiometabolic outcomes, we comprehensively investigated metabolic syndrome, which could have more comprehensive implications on cardiometabolic health. Finally, although the mechanism of the effects of altitude on health is complex, we examined the mediation effects of lifestyle on the correlations. Unlike temperature and hypoxia, lifestyle behaviours are modifiable, so our findings could not only help explain the correlations, but also have public health implications for behavioural interventions. However, the mediating effects should be interpreted cautiously considering their small effect size. Further studies should examine why dietary and PA patterns tend to be healthier and whether and how those patterns can be promoted elsewhere in China and beyond.

## CONCLUSIONS

We found that living at middle and high altitudes was significantly associated with lower risks of metabolic syndrome compared to low altitude, with middle altitude having the lowest risk. We also observed mediation effects of diet and PA. Future studies may examine the promotable healthy parts of dietary and PA patterns at higher altitudes (especially middle altitude).

## Additional material


Online Supplementary Document
