# Supplementary material for: Altitude and metabolic syndrome in China: Beneficial effects of healthy diet and physical activity
Source: J Glob Health. 2023 Jun 30;13:04061. doi: 10.7189/jogh.13.04061 (PMC10312044; doi:10.7189/jogh.13.04061)
Supplement: Online Supplementary Document [file jogh-13-04061-s001.pdf]

Supplementary information

Title: Altitude and Metabolic Syndrome in China: Beneficial Effects of healthy Diet and Physical Activity

Junmin Zhou, PhD[1,#]; Ruifeng He, MD[2,#]; Zhuozhi Shen, MD[3]; Yan Zhang, MD[4]; Xufang Gao, PhD[5]; Dejiquzong, MS[6]; Xiong Xiao, PhD[1]; Tao Zhang, PhD[1]; Dan Yang, MS[1]; Yufei Wang, BS[1]; Huan Song, PhD[7,8]; Yuming Guo, PhD[9]; Shanshan Li， PhD[9]; Gongbo Chen, PhD[10, *], Jianzhong Yin, MS[11,12, *], Xing Zhao, PhD[1,*] On behalf of the China Multi-Ethnic Cohort (CMEC) collaborative group

1 West China School of Public Health and West China Fourth Hospital, Sichuan University, Chengdu, China.
2 Tibet Center for Disease Control and Prevention, Lhasa, China.
3 Chongqing Municipal Center for Disease Control and Prevention, Chongqing, China.
4 School of Public Health, Guizhou Medical University, Guiyang, China.
5 Chengdu Center for Disease Control &Prevention, Chengdu, China.
6 Tibet University, Lhasa, China.
7 West China Biomedical Big Data Center, West China Hospital, Sichuan University, Chengdu 610041, China.
8 Medical Big Data Center, Sichuan University, Chengdu 610041, China.
9 Department of Epidemiology and Preventive Medicine, School of Public Health and Preventive Medicine, Monash University, Melbourne, Australia.
10 Guangzhou Key Laboratory of Environmental Pollution and Health Risk Assessment, Guangdong Provincial Engineering Technology Research Center of Environmental and Health risk Assessment, Department of Preventive Medicine, School of Public Health, Sun Yat-sen University, Guangzhou, China.
11 School of Public Health， Kunming Medical University, Kunming, China.
12 Baoshan College of Traditional Chinese Medicine, Baoshan, China.

[#] These authors contributed equally to this work and share first authorship.

[*] Xing Zhao, Jianzhong Yin, Gongbo Chen share the responsibility of correspondence.

Email: Xing Zhao, xingzhao@scu.edu.cn; Jianzhong Yin, yinjianzhong2005@sina.com; Gongbo Chen, chengb36@mail.sysu.edu.cn

Table of Contents

Supplementary File 1 Explanations for proportion mediated in the mediation analysis

Table S1 Mediation analyses of altitude (binary) and mediators physical activity and dietary pattern (continuous) on metabolic syndrome

Figure S1A
Adjusted risk difference for metabolic syndrome comparing middle altitude group to low altitude group

Figure S1B
Adjusted risk difference for metabolic syndrome comparing high altitude group to low altitude group

Figure S1C
Adjusted risk difference for metabolic syndrome comparing high altitude group to middle altitude group

Table S2 Prevalence of five components of metabolic syndrome, by altitude group

Table S3 Prevalence of possible combinations of metabolic syndrome, by altitude group

Table S4 Correlations between altitude and physical activity, physical activity and metabolic syndrome, altitude and dietary pattern, dietary pattern and metabolic syndrome

Table S5 Baseline characteristics of the Han participants according to different altitude group

Table S6 Prevalence of five components of metabolic syndrome in Han participants, by altitude group

Figure S2 Adjusted risk difference for metabolic syndrome comparing middle altitude group to low altitude group in Han participants

Figure S3 Mediation analyses of altitude (binary) and mediators physical activity and dietary pattern (continuous) on metabolic syndrome in Han participants

Table S7 Mediation analyses of altitude (binary) and mediators physical activity and dietary pattern (continuous) on metabolic syndrome in Han participants

Table S8 Correlations between altitude and physical activity, physical activity and metabolic syndrome, altitude and dietary pattern, dietary pattern and metabolic syndrome in Han participants

Table S9 Prevalence of self-reported chronic diseases, by altitude group

Supplementary File 1 Explanations for proportion mediated in the mediation analysis

1. The definition of proportion mediated in the causal mediation analysis

The approach to Causal mediation analysis was proposed by Imai, et.al. [1,2] R package *mediation* consists of a comprehensive suite of statistical tools has been developed to implement causal mediation analysis.[3] The general framework for the mediation analysis is represented as follow (the text in grey may be skipped for the first read):

Suppose $Y_i(t)$ denote the potential outcome of unit i under the treatment status t (where t = 0, 1), *and* $M_i(t)$ denote the potential value of the mediator for unit i under the treatment status *t*. The causal mediation effects or indirect effects for each unit i are as follow:

$$\delta_i(t) \equiv Y_i\big(t, M_i(1)\big) - Y_i(t, M_i(0)) \tag{1}$$

Similarly, the direct effects of the treatment for each unit i are defined as follows:

$$\xi_i(t) \equiv Y_i\big(1, M_i(t)\big) - Y_i(0, M_i(t)) \tag{2}$$

Then, the total effect of the treatment is decomposed into the causal mediation effects and direct effects:

$$\tau_i(t) \equiv Y_i\big(1, M_i(1)\big) - Y_i\big(0, M_i(0)\big) = \frac{1}{2}\sum_{t=0}^{1}\{\delta_i(t) + \xi_i(t)\} \tag{3}$$

If we add the assumption that there is no interaction between causal mediation effects and direct effects (i.e., $\delta_i = \delta_i(1) = \delta_i(0)$ and $\xi_i = \xi_i(1) = \xi_i(0)$), then the total effect can be simplified as:

$$\tau_i = \delta_i + \xi_i \tag{4}$$

Where δ_i is the mediated (indirect) effect and ξ_i is the direct effect. The proportion mediated, the magnitude of the average causal mediation effects relative to the average total effect, can be defined as:

$$\nu \equiv \frac{\delta}{\tau} \tag{5}$$

which is the ratio of the average causal mediation effects to the average total effect. This proportion-mediated measure can be a helpful summary, as in some sense

it captures how important the pathway through the intermediate is in explaining the actual operation of the effect of the exposure on the outcome.

2. The dilemma of confidence interval of proportion mediated in the causal mediation analysis

The proportion mediated makes sense when the sign of the average causal mediation effects is the same as the sign of the direct effects. It is problematic when the sign of the causal mediation effects and direct effects operate in different directions, which can result in a proportion mediated larger than 100% and such measure may be not meaningful (2nd paragraph, Page 48 [4]).

The R package *mediation* conducts a Monte Carlo experiment to investigate the finite-sample performance of the average causal mediation effect, direct effect and the proportion mediated. Briefly, take a random sample with replacement of size *n* from the original data *J* times. For each of the *J* bootstrapped samples, proportion mediated was computed as mediation effects/(direct effects + mediation effects) (as the equation (5)) for each sample, and then using percentiles for the confidence interval (CI) limits.

Among the *J* bootstrapped samples, if direct effects and mediation effects have opposite signs in a sample, the proportion mediated from this sample is greater than 1(or less than -1). Then the percentile among the *J* bootstrapped samples, which is the confidence interval of proportion mediated, could be outside [0,1]. Similar awkwardness arises in the help document of the “mediation” package; see page 7 of https://cran.r-project.org/web/packages/mediation/vignettes/mediation.pdf.

In our study, the point estimate of proportion mediated was legitimate, as our point estimates of direct and indirect effects were both negative. However, the 95% CI of proportion mediated exceeded 1. In 88% the bootstrapped samples, the direct and mediation effects were both negative, and proportion mediated was reasonably calculated. But in the rest 12% bootstrapped samples, the mediation effects were negative and the direct effects were positive, so the proportion mediated from these sample is larger than 1, resulting in an overall 95% CI with upper limit being

167.14%. It may be a dilemma to define the CI of proportion mediated, but we argue to report a truncated 95% CI with upper limit 100.00%. This CI may still provide some sense of uncertainty.

Reference

[1] Imai K, Keele L, Tingley D. A general approach to causal mediation analysis. Psychol Methods. 2010 Dec;15(4):309-34. doi: 10.1037/a0020761.

[2] Imai K, Keele L and Yamamoto T. Identification, Inference and Sensitivity Analysis for Causal Mediation Effects. 2010;25 J Statistical Science:51-71, 21. doi: 10.1214/10-STS321.

[3] Tingley D, Yamamoto T, Hirose K, Keele L, Imai K. Mediation: R Package for Causal Mediation Analysis. Journal of Statistical Software. 2014: 17978. doi: 10.18637/jss.v059.i05.

[4] VanderWeele TJ. Explanation in causal inference: methods for mediation and interaction. Oxford: Oxford University Press; 2015.

Table S1 Mediation analyses of altitude (binary) and mediators physical activity and dietary pattern (continuous) on metabolic syndrome

Exposures	Potential mediators	Mediation effect (%) (95% CI)	Proportion mediated (%) (95% CI)	*P* value
Middle to Low	Physical activity	-0.94 (-1.04, -0.86)	26.10 (22.05, 34.92)	<0.0001
	Dietary pattern	-0.40 (-0.47, -0.32)	11.62 (8.30, 14.94)	<0.0001
High to Low	Dietary pattern	-0.72 (-0.87, -0.58)	52.19 (26.38, 167.14)	<0.0001

Analyses were adjusted for age, sex, marital status, education, income, smoking status, passive smoking, and alcohol consumption (continuous). CI, confidence interval.

Figure S1A

Adjusted risk difference for metabolic syndrome comparing middle altitude group to low altitude group

Middle to Low Altitude

Subgroup	No. of Participants	Risk difference (95%CI)
**Age (yr)**		
30-60	66,424	-2.25 (-3.03, -1.52)
60-70	16,668	-7.26 (-8.83, -5.56)
70-79	6,393	-8.55 (-11.44, -5.54)
Trend χ^2=1259.63; P<0 .0001		
**Sex**		
Male	35,295	-5.8 (-6.95, -4.66)
Female	54,190	-2.65 (-3.47, -1.87)
Heterogeneity χ^2=18.65; P=<0.0001		
**Marital status**		
Did not cohabit	10,041	-4.23 (-6.3, -2.01)
Cohabited	79,444	-3.45 (-4.12, -2.72)
Heterogeneity χ^2=0.46; P=0.50		
**Education**		
Illiteracy	23,746	-3.83 (-5.18, -2.56)
Primary school	22,688	-4.45 (-5.63, -3.32)
Middle school	22,903	-5.36 (-6.6, -4.11)
High school or above	20,148	-1.76 (-3.67, 0.42)
Trend χ^2=197.68; P=<0.0001		
**Income (RMB)**		
<20,000	31,946	-3.1 (-4.12, -2.1)
20,000 – 59,999	32,324	-5.15 (-6.17, -4.16)
≥ 60,000	25,215	-1.56 (-3.19, 0.04)
Trend χ^2=1.56; P=0.2114		
**Smoking status**		
Never	67,227	-2.38 (-3.07, -1.62)
Current	17,752	-7.51 (-8.97, -5.89)
Former	4,506	-5.47 (-9.05, -1.54)
Heterogeneity χ^2=35.89; P<0.0001		
**Passive smoking**		
No	46,398	-3.14 (-4.09, -2.24)
Yes	43,087	-3.72 (-4.61, -2.82)
Heterogeneity χ^2=0.74; P=0.39		
**Alcohol consumption (g/d)**		
0	51,643	-3.34 (-4.17, -2.51)
(0, 5)	26,656	-4.11 (-5.61, -2.63)
≥5	11,186	-7.61 (-9.56, -5.58)
Trend χ^2=23.74; P=<0.0001		
**Overall**	**89,485**	**-3.54 (-4.24, -2.86)**
Heterogeneity χ^2=142.56; P=<0.0001		

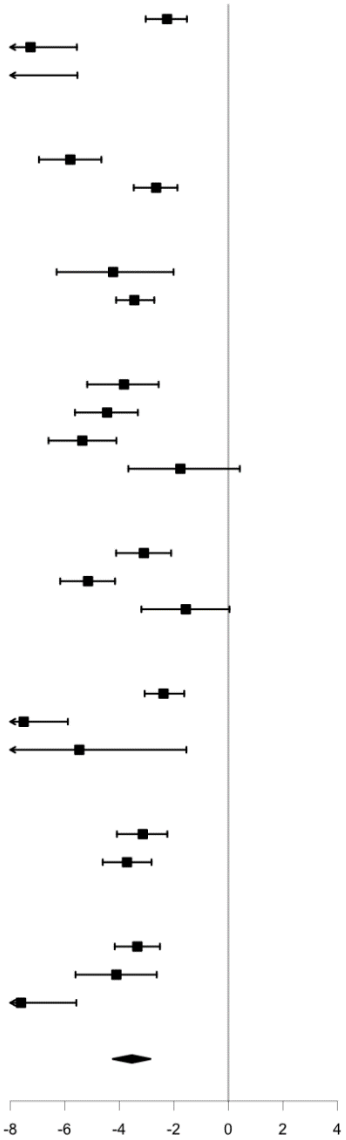

Figure S1B

Adjusted risk difference for metabolic syndrome comparing high altitude group to low altitude group

High to Low Altitude

Subgroup	No. of Participants	Risk difference (95%CI)
**Age (yr)**		
30-60	66,424	-4.23 (-5.22, -3.09)
60-70	16,668	-5.2 (-7.87, -2.19)
70-79	6,393	-4.92 (-10.09, 0.24)
Trend χ^2=1251.17; P<0.0001		
**Sex**		
Male	35,295	3.94 (1.89, 5.98)
Female	54,190	-3.54 (-4.84, -2.21)
Heterogeneity χ^2=37.40; P=<0.0001		
**Marital status**		
Did not cohabit	10,041	-4.58 (-7.55, -1.56)
Cohabited	79,444	-1.18 (-2.26, -0.04)
Heterogeneity χ^2=3.92; P=0.04		
**Education**		
Illiteracy	23,746	-5.61 (-7.11, -4.08)
Primary school	22,688	3.35 (1.3, 5.52)
Middle school	22,903	2.37 (-1.42, 6.39)
High school or above	20,148	4.7 (0, 9.08)
Trend χ^2=101.33; P=<0.0001		
**Income (RMB)**		
<20,000	31,946	-1.31 (-2.82, 0.24)
20,000 – 59,999	32,324	-3.44 (-5.06, -1.71)
≥ 60,000	25,215	0.09 (-2.51, 2.72)
Trend χ^2=1.20; P=0.2743		
**Smoking status**		
Never	67,227	-3.15 (-4.24, -2.07)
Current	17,752	4.92 (1.63, 8.32)
Former	4,506	7.19 (1.61, 12.91)
Heterogeneity χ^2=30.33; P<0.0001		
**Passive smoking**		
No	46,398	-1.98 (-3.14, -0.78)
Yes	43,087	0.66 (-1.77, 3.22)
Heterogeneity χ^2=3.47; P=0.06		
**Alcohol consumption (g/d)**		
0	51,643	-3.27 (-4.36, -2.07)
(0, 5)	26,656	5.77 (3.1, 8.29)
≥5	11,186	4.11 (-1.1, 9.6)
Trend χ^2=52.06; P=<0.0001		
**Overall**	**89,485**	**-1.53 (-2.53, -0.46)**
Heterogeneity χ^2 =209.45; P=<0.0001		

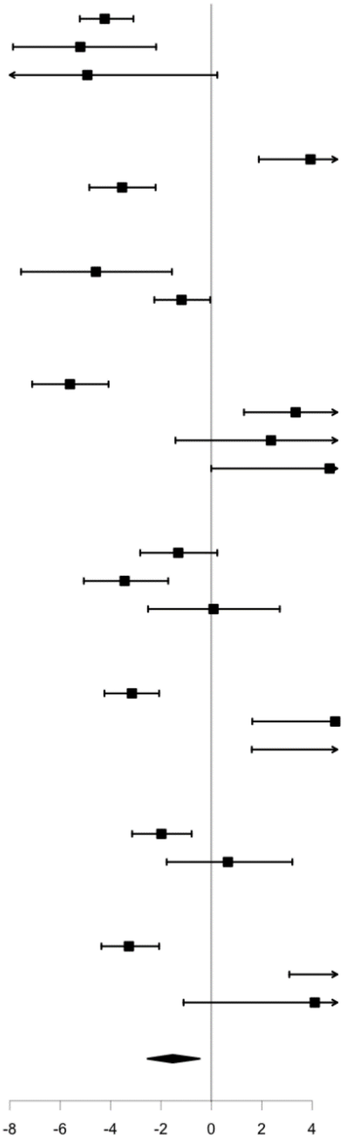

Figure S1C

Adjusted risk difference for metabolic syndrome comparing high altitude group to middle altitude group

High to Middle Altitude

Subgroup	No. of Participants	Risk difference (95%CI)
**Age (yr)**		
30-60	66,424	-1.97 (-3.04, -0.79)
60-70	16,668	2.07 (-0.81, 5.3)
70-79	6,393	3.63 (-2.41, 9.27)
Trend χ^2=193.08; P<0.0001		
**Sex**		
Male	35,295	9.74 (7.5, 11.83)
Female	54,190	-0.89 (-2.19, 0.4)
Heterogeneity χ^2=68.64; P=<0.0001		
**Marital status**		
Did not cohabit	10,041	-0.35 (-3.85, 3.01)
Cohabited	79,444	2.27 (1.1, 3.37)
Heterogeneity χ^2=2.01; P=0.16		
**Education**		
Illiteracy	23,746	-1.78 (-3.45, -0.04)
Primary school	22,688	7.8 (5.68, 9.93)
Middle school	22,903	7.73 (3.64, 11.89)
High school or above	20,148	6.45 (1.41, 11.24)
Trend χ^2=14.82; P=0.0001		
**Income (RMB)**		
<20,000	31,946	1.79 (0.22, 3.49)
20,000 – 59,999	32,324	1.71 (0.03, 3.46)
≥ 60,000	25,215	1.65 (-1.16, 4.42)
Trend χ^2=2.63; P=0.1046		
**Smoking status**		
Never	67,227	-0.77 (-2.01, 0.46)
Current	17,752	12.43 (9.05, 15.68)
Former	4,506	12.66 (6.24, 18.66)
Heterogeneity χ^2=63.51; P<0.0001		
**Passive smoking**		
No	46,398	1.17 (-0.11, 2.59)
Yes	43,087	4.37 (1.99, 7.07)
Heterogeneity χ^2=4.80; P=0.03		
**Alcohol consumption (g/d)**		
0	51,643	0.07 (-1.11, 1.38)
(0, 5)	26,656	9.89 (7.35, 12.54)
≥5	11,186	11.72 (6.3, 17.28)
Trend χ^2=2.66; P=0.1030		
**Overall**	**89,485**	**2.01 (0.92, 3.09)**
Heterogeneity χ^2 =290.90; P=<0.0001		

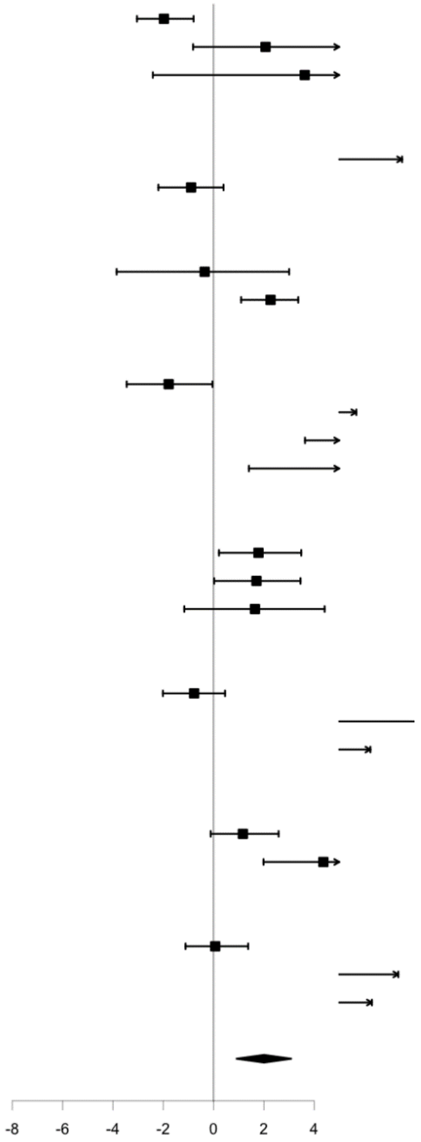

Analyses were adjusted for age, sex, marital status, education, income, smoking status, passive smoking, alcohol consumption, physical activity, and dietary pattern and were stratified according to covariates. The black boxes represent risk differences, and the horizontal lines represent 95% confidence intervals. The diamonds indicate the overall risk differences and the 95% confidence intervals. Chi-square tests (χ^2) were conducted to examine either trend (with 1 df) or heteogeneity (with n-1 df, where n represents the number of categories).

Table S2 Prevalence of five components of metabolic syndrome, by altitude group

Components of metabolic syndrome (%)	High altitude		Middle altitude		Low altitude		*P* value
	Prevalence	95% CI	Prevalence	95% CI	Prevalence	95% CI	
Elevated waist circumference	53.36	(52.44, 54.27)	24.52	(23.96, 25.08)	31.56	(31.20, 31.92)	<0.001
Elevated triglycerides	19.75	(19.02, 20.48)	36.80	(36.17, 37.43)	35.11	(34.74, 35.49)	<0.001
Reduced HDL-C	27.06	(26.24, 27.87)	18.84	(18.34, 19.35)	20.75	(20.43, 21.06)	<0.001
Elevated BP	33.27	(32.41, 34.14)	45.00	(44.36, 45.65)	45.23	(44.85, 45.62)	<0.001
Elevated fasting glucose	4.17	(3.80, 4.54)	23.30	(22.75, 23.85)	25.21	(24.87, 25.54)	<0.001

CI, confidence interval.

Table S3 Prevalence of possible combinations of metabolic syndrome, by altitude group

Possible combinations of metabolic syndrome (%)	High altitude		Middle altitude		Low altitude		*P* value
	Prevalence	95% CI	Prevalence	95% CI	Prevalence	95% CI	
Elevated waist circumference & Elevated triglycerides & Reduced HDL-C	5.97	(5.54, 6.40)	6.20	(5.89, 6.51)	6.58	(6.39, 6.77)	<0.001
Elevated waist circumference & Elevated triglycerides & Elevated BP	7.53	(7.05, 8.02)	9.19	(8.81, 9.56)	11.21	(10.96, 11.45)	<0.001
Elevated waist circumference & Elevated triglycerides & Elevated fasting glucose	1.64	(1.41, 1.88)	5.20	(4.91, 5.49)	7.40	(7.20, 7.61)	<0.001
Elevated waist circumference & Reduced HDL-C & Elevated BP	7.30	(6.82, 7.77)	4.69	(4.41, 4.96)	5.59	(5.41, 5.77)	<0.001
Elevated waist circumference & Reduced HDL-C & Elevated fasting glucose	1.36	(1.15, 1.58)	2.74	(2.53, 2.95)	3.85	(3.70, 3.99)	<0.001
Elevated waist circumference & Elevated BP & Elevated fasting glucose	0	(0, 0)	0	(0, 0)	0	(0, 0)	-
Elevated triglycerides & Reduced HDL-C & Elevated BP	3.43	(3.10, 3.77)	7.95	(7.60, 8.30)	6.68	(6.49, 6.88)	-
Elevated triglycerides & Reduced HDL-C & Elevated fasting glucose	0	(0, 0)	0	(0, 0)	0	(0, 0)	-
Elevated triglycerides & Elevated BP & Elevated fasting glucose	1.23	(1.03, 1.43)	7.57	(7.22, 7.91)	9.08	(8.86, 9.31)	<0.001

Reduced HDL-C & Elevated BP & Elevated fasting glucose	0.96	(0.78, 1.14)	3.47	(3.23, 3.71)	4.14	(3.99, 4.30)	<0.001

CI, confidence interval.

Table S4 Correlations between altitude and physical activity, physical activity and metabolic syndrome, altitude and dietary pattern, dietary pattern and metabolic syndrome

	Coefficient	95% CI
	Altitude → Physical activity	
Altitude		
Middle to Low	6.62	(6.34, 6.90)
High to Low	-8.14	(-8.56, -7.71)
High to Middle	-14.76	(-15.21, -14.31)
	Physical activity → Metabolic syndrome	
Physical activity (continuous)	-0.0081	(-0.0090, -0.0072)
	Altitude → Dietary pattern	
Altitude		
Middle to Low	1.10	(1.04, 1.17)
High to Low	1.97	(1.87, 2.08)
High to Middle	0.87	(0.76, 0.98)
	Dietary pattern → Metabolic syndrome	
Dietary pattern (DASH score)	-0.020	(-0.024, -0.016)

Analyses were adjusted for age, sex, marital status, education, income, smoking status, passive smoking, and alcohol consumption (continuous). CI, confidence interval.

Table S5 Baseline characteristics of the Han participants according to different altitude group

Characteristic	All participants (N=52,181)	Altitude		*P* value
		Middle altitude (N=9,742)	Low altitude (N=42,439)	
Age	51.55±11.72	52.54±10.25	51.33±12.02	< 0.001
Sex (%)				< 0.001
Male	43.31	33.78	45.40	
Female	56.69	66.22	54.60	
Marital status (%)				< 0.001
Did not cohabit	10.87	10.19	11.06	
Cohabited	89.13	89.81	88.94	
Educational level (%)				< 0.001
Illiteracy	13.79	25.70	10.67	
Primary school	24.46	36.25	21.82	
Junior high school	31.01	27.53	32.50	
High school or above	30.75	10.52	35.02	
Income (yuan, %)*				< 0.001
< 20,000	26.41	42.00	22.78	
20,000 – 59,999	37.16	43.92	35.90	
≥ 60,000	36.43	14.08	41.32	
Smoking status (%)				< 0.001
Never	71.47	75.53	70.55	
Current	22.56	21.44	22.77	
Former	5.97	3.03	6.68	
Passive smoking (%)				< 0.001
No	47.44	43.72	48.28	
Yes	52.56	56.28	51.72	
Alcohol consumption (g/d)				< 0.001
0	51.53	71.13	47.17	
(0, 5)	33.28	17.16	36.81	
≥ 5	15.19	11.71	16.02	
Physical activity (MET-h/day)†	24.87±17.45	34.92±19.54	22.57±15.40	< 0.001
Dietary pattern (Dash score)	21.41±4.41	21.45±3.72	21.40±4.53	0.003
Metabolic syndrome				< 0.001
No	74.65	80.97	73.21	
Yes	25.35	19.03	26.79	

Plus-minus values are means±SD. Data were age adjusted where appropriate.

* As of January 2021, the exchange rate was approximate 6.46 Yuan per U.S. dollar.

† MET denotes metabolic equivalent.

Table S6 Prevalence of five components of metabolic syndrome in Han participants, by altitude group

Components of metabolic syndrome (%)	Middle altitude		Low altitude		*P* value
	Prevalence	95% CI	Prevalence	95% CI	
Elevated waist circumference	16.30	(15.59, 17.01)	30.80	(30.37, 31.23)	<0.001
Elevated triglycerides	40.73	(39.78, 41.67)	34.49	(34.05, 34.93)	<0.001
Reduced HDL-C	23.10	(22.29, 23.91)	22.29	(21.90, 22.67)	0.024
Elevated BP	38.80	(37.86, 0.397)	46.37	(45.91, 46.83)	<0.001
Elevated fasting glucose	15.06	(14.37, 15.74)	25.54	(25.14, 25.95)	<0.001

CI, confidence interval.

Figure S2 Adjusted risk difference for metabolic syndrome comparing middle altitude group to low altitude group in Han participants

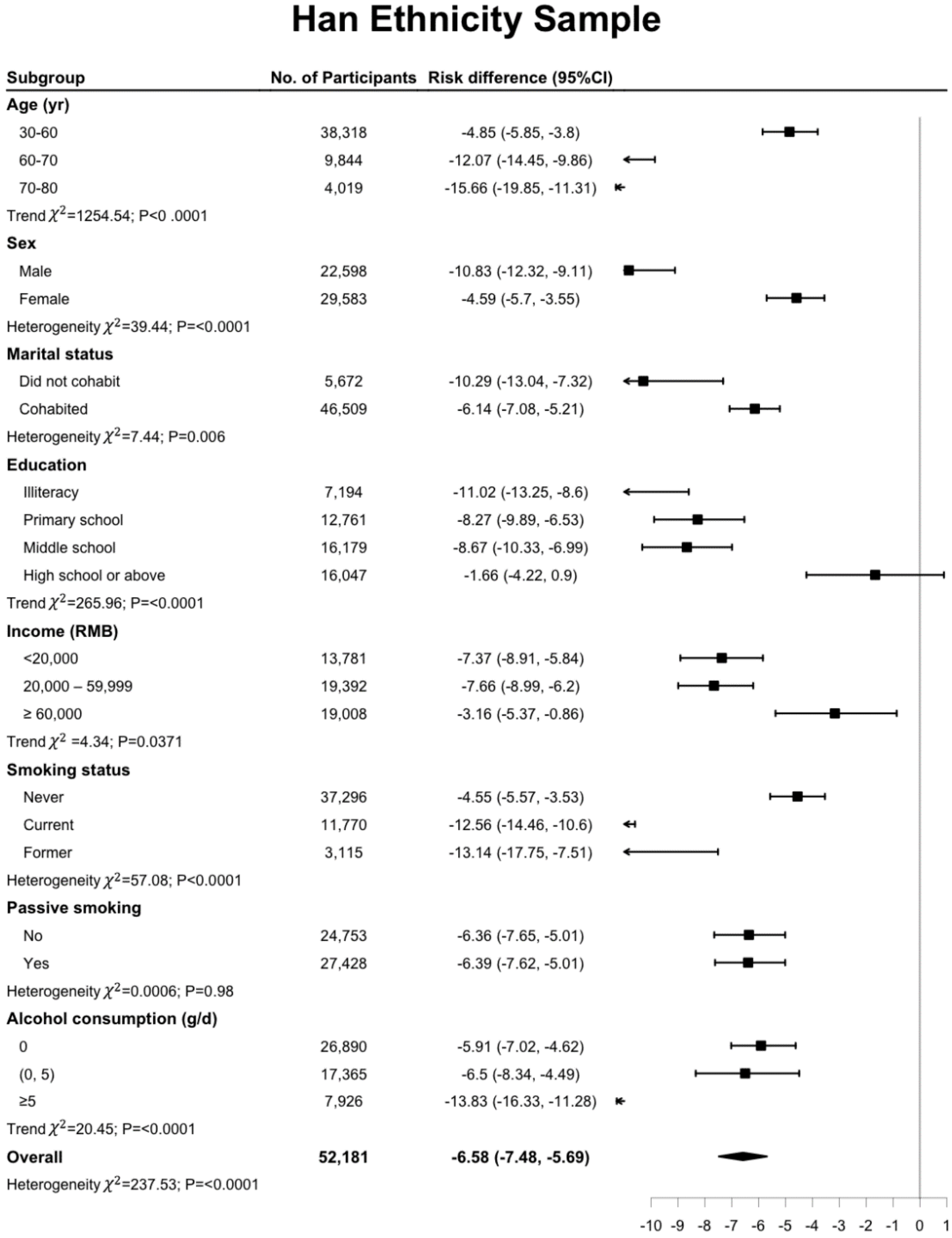

Analyses were adjusted for age, sex, marital status, education, income, smoking status, passive smoking, alcohol consumption, physical activity, and dietary pattern and were stratified according to covariates. The black boxes represent risk differences, and the horizontal lines represent 95% confidence intervals. The diamonds indicate the overall risk differences and the 95% confidence intervals. Chi-square tests (χ^2) were conducted to examine either trend (with 1 df) or heteogeneity (with n-1 df, where n represents the number of categories).

Figure S3 Mediation analyses of altitude (binary) and mediators physical activity and dietary pattern (continuous) on metabolic syndrome in Han participants

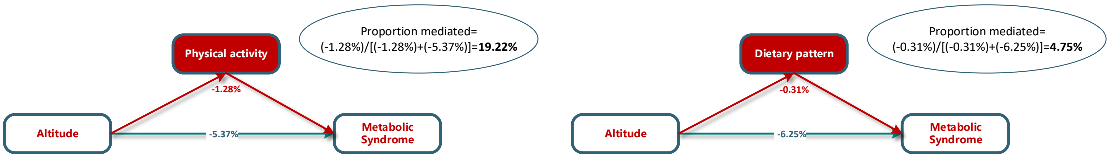

a. Middle altitude VS. Low altitude, physical activity as the mediator

b. Middle altitude VS. Low altitude, dietary pattern as the mediator

Analyses were adjusted for age, sex, marital status, education, income, smoking status, passive smoking, and alcohol consumption.

Table S7 Mediation analyses of altitude (binary) and mediators physical activity and dietary pattern (continuous) on metabolic syndrome in Han participants

Exposures	Potential mediators	Mediation effect (%) (95% CI)	Proportion mediated (%) (95% CI)	*P* value
Middle to Low	Physical activity	-1.28 (-1.51, -1.02)	19.22 (14.55, 24.30)	<0.0001
	Dietary pattern	-0.31 (-0.37, -0.24)	4.75 (3.40, 5.85)	<0.0001

Analyses were adjusted for age, sex, marital status, education, income, smoking status, passive smoking, and alcohol consumption. CI, confidence interval.

Table S8 Correlations between altitude and physical activity, physical activity and metabolic syndrome, altitude and dietary pattern, dietary pattern and metabolic syndrome in Han participants

	Coefficient	95% CI
	Altitude → Physical activity	
Altitude		
Middle to Low	10.00	(9.62, 10.37)
	Physical activity → Metabolic syndrome	
Physical activity (continuous)	-0.0075	(-0.0088, -0.0062)
	Altitude → Dietary pattern	
Altitude		
Middle to Low	0.92	(0.83, 1.02)
	Dietary pattern → Metabolic syndrome	
Dietary pattern (DASH score)	-0.020	(-0.025, -0.015)

Analyses were adjusted for age, sex, marital status, education, income, smoking status, passive smoking, and alcohol consumption. CI, confidence interval.

Table S9 Prevalence of self-reported chronic diseases, by altitude group

Chronic disease (%)	All participants (N=89,485)	Altitude			*P* value
		High altitude (N=8,701)	Middle altitude (N=21,395)	Low altitude (N=59,389)	
Hypertension					<0.001
No	82.89	83.13	81.08	83.51	
Yes	17.11	16.87	18.92	16.49	
Diabetes					<0.001
No	95.17	97.76	97.02	94.12	
Yes	4.83	2.24	2.98	5.88	
Hyperlipidemia					<0.001
No	92.34	87.45	95.59	91.89	
Yes	7.66	12.55	4.41	8.11	
Chronic Bronchitis/Emphysema					0.051
No	93.62	93.56	93.28	93.76	
Yes	6.38	6.44	6.72	6.24	
Chronic Hepatitis/Cirrhosis					<0.001
No	97.24	94.70	99.20	96.91	
Yes	2.76	5.30	0.80	3.09	
Pulmonary heart disease					0.85
No	99.60	99.61	99.58	99.61	
Yes	0.40	0.39	0.42	0.39	
Rheumatic heart disease					<0.001
No	99.78	99.38	99.90	99.80	
Yes	0.22	0.62	0.10	0.20	
Coronary heart disease					<0.001
No	97.40	97.46	98.64	96.95	
Yes	2.60	2.54	1.36	3.05	
Rheumatoid arthritis					<0.001
No	93.17	90.86	90.49	94.48	
Yes	6.83	9.14	9.51	5.52	
Asthma					<0.001
No	98.61	98.89	99.13	98.38	
Yes	1.39	1.11	0.87	1.62	
Gallstones/Cholecystitis					<0.001
No	88.03	75.72	91.52	88.58	
Yes	11.97	24.28	8.48	11.42	
Peptic ulcers					<0.001
No	97.15	96.21	98.33	96.86	
Yes	2.85	3.79	1.67	3.14	

Gastroenteritis					<0.001
No	87.53	82.91	87.72	88.14	
Yes	12.47	17.09	12.28	11.86	
